# Predictive criteria of severe cases in COVID‐19 patients of early stage: A retrospective observational study

**DOI:** 10.1002/jcla.23562

**Published:** 2020-09-06

**Authors:** Jinrui Gao, Xiu Huang, Haibo Gu, Lingyun Lou, Zhihao Xu

**Affiliations:** ^1^ Department of Respiratory and Critical Care Medicine Fourth Affiliated Hospital of Zhejiang University School of Medicine Yiwu China

**Keywords:** COVID‐19, cytokine storm, laboratory parameter, risk factors, SARS‐CoV‐2

## Abstract

**Background:**

Patients with coronavirus disease 2019 (COVID‐19) often suffer sudden deterioration of disease around 1‐2 weeks after onset. Once the disease progressed to severe phase, clinical prognosis of patients will significantly deteriorate.

**Methods:**

This was a multicenter retrospective study on patients of all adult inpatients (≥18 years old) from Tianyou Hospital (Wuhan, China) and the Fourth Affiliated Hospital, Zhejiang University School of Medicine. All 139 patients had laboratory‐confirmed COVID‐19 in their early stage, which is defined as within 7 days of clinical symptoms. Univariate and multivariate logistic regression models were used to determine the predictive factors in the early detection of patients who may subsequently develop into severe cases.

**Results:**

Multivariable logistic regression analysis showed that the higher level of hypersensitivity C‐reactive protein (OR = 4.77, 95% CI:1.92‐11.87, *P* = .001), elevated alanine aminotransferase (OR = 6.87, 95%CI:1.56‐30.21, *P* = .011), and chronic comorbidities (OR = 11.48, 95% CI:4.44‐29.66, *P* < .001) are the determining risk factors for the progression into severe pneumonia in COVID‐19 patients.

**Conclusion:**

Early COVID‐19 patients with chronic comorbidities, elevated hs‐CRP or elevated ALT are significantly more likely to develop severe pneumonia as the disease progresses. These risk factors may facilitate the early diagnosis of critical patients in clinical practice.

## Graphical abstract

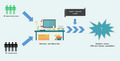

## 1 INTRODUCTION

Coronavirus disease 2019 (COVID‐19) is an acute respiratory infectious disease caused by severe and highly contagious acute respiratory syndrome coronavirus 2 (SARS‐CoV‐2; the *Coronaviridae* family). Although previous studies suggested that the basic reproduction number (R0) of SARS‐CoV‐2 was between 2 and 3, the countries with prompt isolation were successful in reducing the reproduction number.^1^ Extensive research has shown that the total mortality rate is 5.6%, lower than SARS (13%) and MERS (35%).^2^ But the death toll has far exceeded. As of August 7, 2020, the world had 19 282 031 confirmed cases of and 718 074 deaths from COVID‐19, according to publicly available data from Worldometer website.

COVID‐19 patients can suddenly worsen around 1‐2 weeks after the onset of the disease. It has previously been observed that the time from the onset of symptoms to dyspnea is 8.0 days (IQR: 5.0 ‐ 13.0 days).^3^ Once the disease progressed to a severe stage, the prognosis will be extremely poor, achieving a booming mortality rate of as high as 49.0%.^4^ At the same time, recent evidence suggests that 77% of severe cases need to be admitted to ICU, while mild patients do not have such need.^5^ Therefore, an early identification and timely intervention of patients with tendency to become severe and critical condition is critical to improve clinical prognosis and save medical resources. What is less clear, however, is how to identify patients with severe tendency during the early stage of COVID‐19.

The objective of this study was to determine the predictive criteria of severe tendency of early COVID‐19 patients by systematically analyzing the baseline clinical characteristics and laboratory indexes of severe patients and non‐severe patients. Results from this study will aid in the development of an effective measures tool for the early clinical identification and intervention of severe cases in the COVID‐19 outbreak.

## 2 PATIENTS AND METHODS

### 2.1 Patients and data collection

This retrospective study enrolled a total of 139 adult inpatients with confirmed COVID‐19 from Tianyou Hospital (Wuhan, China) and the Fourth Affiliated Hospital, Zhejiang University School of Medicine. All patients received complete medical history collection, vital signs measurement, and laboratory and radiological examinations. The laboratory and radiological data included, but were not limited to, lymphocyte counts, hypersensitive C‐reactive protein (hs‐CRP), concentrations of D‐dimer, erythrocyte sedimentation rate (ESR), prothrombin time (PT), procalcitonin (PCT), alanine transaminase (ALT), high‐sensitivity troponin T (hs‐TNT), and chest CT images. Related data were collected and cross‐checked by two doctors to assure the data quality. To avoid the effects and side effects of drugs on laboratory parameters, the laboratory data used in our study are from early COVID‐19 patients basically before medication. COVID‐19 patients of early stage are defined as within 7 days of clinical symptoms.

### 2.2 Assessment and grouping

Severity of the disease was staged according to the guidelines for diagnosis and treatment of COVID‐19 (trial seventh edition) published by the National Health Commission of China on March 4, 2020. Patients who had any of the following features at the time of, or after admission were classified as severe cases: (a) respiratory distress (≥30 breaths per minute); (b) oxygen saturation at rest ≤ 93%; (c) oxygenation index (artery partial pressure of oxygen/inspired oxygen fraction, PaO_2_/FiO_2_) ≤300 mm Hg; and (d) Lung imaging showed that the lesion progressed more than 50% within 24 hours and 48 hours. As a result, there were 93 cases in the severe group and 46 cases in the non‐severe group.

### 2.3 Statistical analysis

Statistical analyses were performed using SPSS 20.0 software. Measurement data were described by median and interquartile ranges (IQR). Differences of measurement data were compared with Mann‐Whitney *U* test. The enumeration data were represented by frequencies and percentages. To compare the enumeration data of different groups, chi‐square and Fisher's exact test were used. Univariate and multivariate logistic regression models were used to analyze the risk factors for severe tendency of early COVID‐19 patients. *P* < .05 was considered statistically significant.

## 3 RESULTS

### 3.1 General clinical data of the COVID‐19 patients

As indicated in Table 1, the median age of patients was 60 years (IQR, 47‐69; range from 19 to 89 years). The age of the patients in the severe group was older than that in the non‐severe group (*P* < .001). 52% of the patients are male, while no significant correlation between sex and severity of the disease (*P* = .059) was found. 76 patients were complicated with chronic comorbidities, of which hypertension (32%) was the most common, followed by chronic obstructive pulmonary disease (17%) and diabetes (15%). There was no significant difference in antiviral therapy, antibiotic therapy, oxygen therapy, and traditional Chinese medicine treatment between two groups (*P* > .05), but patients in the severe group were more likely to receive glucocorticoid therapy (*P* = .012).

**Table 1:** Baseline characteristics of patients with COVID‐19

	Total (n = 139)	Non‐severe (n = 46)	Severe (n = 93)	*P* Value
Age (year),median (IQR)	60.0 (47.0‐69.0)	48.0 (34.0‐63.0)	62.0 (53.0‐69.0)	<.001
Sex				
Female	67 (48%)	27 (59%)	40 (43%)	.059
Male	72 (52%)	19 (41%)	53 (57%)	
Chronic comorbidities	76 (55%)	11 (24%)	65 (70%)	<.001
Hypertension	42 (30%)	7 (15%)	35 (38%)	.005
Diabetes	21 (15%)	1 (2%)	20 (22%)	.001
Coronary artery disease	16 (12%)	3 (7%)	13 (14%)	.26
COPD	23 (17%)	3 (7%)	20 (22%)	.029
other	12 (9%)	3 (7%)	9 (10%)	.57
Treatment				
Glucocorticoid therapy	22 (16%)	2 (4%)	20 (22%)	.012
Antiviral treatment	82 (59%)	32 (70%)	50 (54%)	.10
Antibiotic therapy	45（32%）	10 (22%)	35 (38%)	.082
Oxygen therapy	139（100%）	46 (100%)	93 (100%)	1
Traditional Chinese medicine therapy	139（100%）	46 (100%)	93 (100%)	1

Note

Data are median (IQR), n (%), or n/N (%). *P* values were calculated by Mann‐Whitney U test, chi‐square test, or Fisher's exact test, as appropriate.

Abbreviation: COPD, chronic obstructive lung disease.

### 3.2 Radiological and laboratory results

Lymphocyte count was significantly lower in severe group than the non‐severe group (0.93 vs 1.2, *P* = .001), while NLR (neutrophil‐to‐lymphocyte ratio) was higher (4.48 vs 2.54, *P* = .01) (Table 2). In addition, the severe group showed the significantly higher levels of hs‐CRP (*P* < .001), D‐dimer (*P* < .035), and ALT (*P* < .001). Although a statistical difference was observed in the level of LDH between the two groups (*P* = .01), if we take the upper limit of normal value (250U/L) as a standard, the difference was insignificant (*P* = .33). Lung lesions in the chest CT images were detected for all patients. The imaging manifestation of severe patients and non‐severe patients were different (*P* < .001). In the severe group, bilateral lung lesions were more common (92%).

**Table 2:** Laboratory results and CT characteristics of COVID‐19 patients in 2 groups

	Total (n = 139)	Non‐severe (n = 46)	Severe (n = 93)	*P* Value
WBC (×10^9^/L)	5.6 (2.9‐8.6)	4.98 (3.3‐6.3)	5.9 (3.0‐8.7)	.062
<4	39 (28%)	15 (32%)	24 (26%)	.62
4‐10	96 (69%)	30 (65%)	66 (71%)	
>10	4 (3%)	1 (2%)	3 (3%)	
Lymphocyte count (x10^9^/L)	1.01 (0.77‐1.4)	1.2 (0.94‐1.6)	0.93 (0.60‐1.3)	.001
<0.8	39 (28%)	6 (13%)	33 (35%)	.004
NLR	3.39 (2.18‐6.24)	2.54 (1.80‐3.41)	4.48 (2.48‐7.13)	.001
Anemia	10 (7%)	4 (9%)	6 (6%)	.43
ESR (mm/h)	38.5 (21.8‐60.5)	39.1 (26.2‐56.4)	38.5 (20.8‐64)	.349
Hs‐CRP (mg/L)	26.8 (5.1‐46.4)	5.05 (1.4‐27.25)	35.1 (12.8‐60.1)	<.001
<10	56 (40%)	28 (61%)	28 (30%)	<.001
10‐50	53 (38%)	16 (35%)	37 (40%)	
>50	30 (22%)	2 (4%)	28 (30%)	<.001
PT (s)	12.0 (11.3‐12.6)	10.9 (11.6‐12.3)	12.2 (11.6‐13.1)	.052
>16	7 (5%)	1 (2%)	6 (6%)	.26
D‐dimer (μg/mL)	0.5 (0.08‐1.4)	0.20 (0.01‐0.57)	0.67 (0.15‐1.80)	.035
>0.5	47 (34%)	9 (20%)	38 (41%)	.009
CK ( U/L)	60 (35.5‐133)	54 (34‐120)	61 (37‐155)	.78
LDH (U/L)	243 (194‐314)	212 (162‐266)	274 (219‐369)	.01
>250	41 (29%)	11 (24%)	30 (32%)	.33
Creatinine (>110μmol/L)	7 (5%)	1 (2%)	6 (6%)	.43
ALT (U/L)	24 (18‐46)	19.5 (14‐28)	27 (20‐54)	<.001
>50U/L	22 (16%)	3 (6%)	19 (20%)	.026
Hs‐TNT (ng/mL)	0.004 (0.00‐0.015)	0.004 (0.00‐0.008)	0.005 (0.00‐0.021)	.166
PCT (ng/mL)	0.03 (0.01‐0.04)	0.029 (0.01‐0.04)	0.03 (0.02‐0.045)	.316
CT positive				
Bilateral lungs	114 (82%)	28 (61%)	86 (92%)	<.001

Note

Data are median (IQR), n (%), or n/N (%). *P* values were calculated by Mann‐Whitney U test, chi‐square test, or Fisher's exact test, as appropriate.

Abbreviations: ALT, alanine transaminase; CK creatine kinase; ESR, erythrocyte sedimentation rate; hs‐CRP, hypersensitive C‐reactive protein; hs‐TNT, high‐sensitivity troponin T; LDH, lactate dehydrogenase; PCT, procalcitonin; PT, prothrombin time; WBC, white blood cell count.

### 3.3 Risk factors for the progression into severe cases

**Figure 1:**
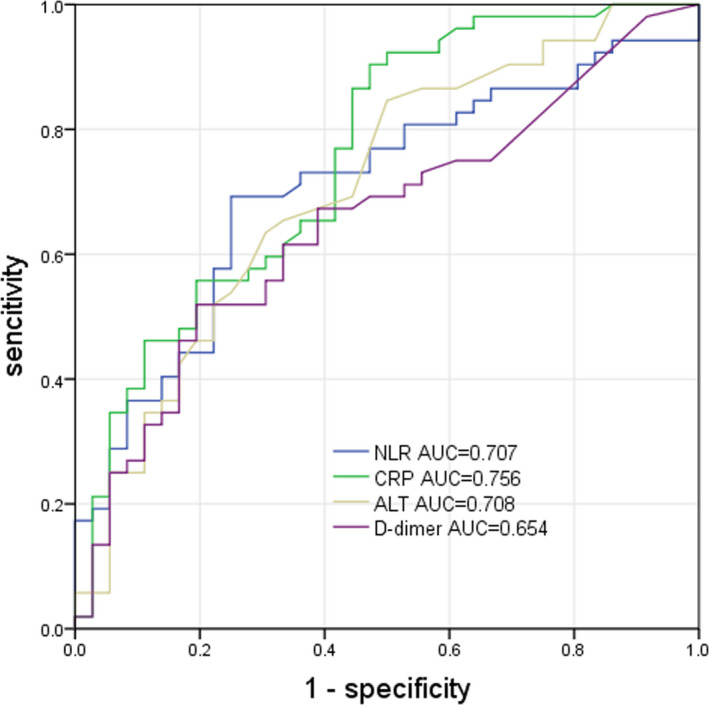
ROC curve for hs‐CRP, D‐dimer, ALT, and NLR

Through univariate logistic regression models, we found that advanced age (≥65 years old), chronic comorbidities, lymphocytopenia, elevated hs‐CRP, increased D‐dimer, and elevated levels of ALT were the key risk factors for the progression of COVID‐19 patients into their severe stage (Table 3). And then we analyzed the optimal cutoff values calculated by the ROC analysis, and the ROC curves were presented in Figure 1. Areas under the curve (AUC) of hs‐CRP, D‐dimer, ALT, and NLR are 0.756 (95%CI: 0.652‐0.860, *P* < .001), 0.654 (95%CI: 0.539‐0.769, *P* = .015), 0.708 (95%CI: 0.597‐0.819 *P* = .001), and 0.707 (95%CI: 0.598‐0.819, *P* = .001). The optimal cutoff value was 3.73 for NLR with sensitivity of 69.2% and specificity of 75.0%. And the risk of COVID‐19 was found to be 4.62‐fold greater when NLR was ≥ 3.73 in the univariate logistic regression (*P* < .007).

**Table 3:** Risk factors associated with severe cases of COVID‐19 patients

	Univariate analysis		Multivariate analysis	
	OR (95% CI)	*P* value	OR (95% CI)	*P* value
Age (<65 y vs ≥65 y)	2.72 (1.21‐6.12)	.015		
Chronic comorbidities (yes vs no)	7.39 (3.28‐16.60)	<.001	11.48 (4.44‐29.66)	<.001
Hypertension	3.36 (1.36‐8.33)	.005	3.36 (1.41‐9.52)	.008
Diabetes	12.33 (1.59‐95.05)	.01	16.53 (2.07‐132.3)	.008
COPD	3.92 (1.10‐13.99)	.006	4.53 (1.21‐16.93)	.025
Coronary artery disease	2.07 (0.42‐10.17)	.26		
Lymphocyte count (<0.8 × 10^9^/L vs ≥ 0.8 × 10^9^/L)	3.67 (1.41‐9.55)	.038		
Hs‐CRP (mg/L)				
<10 vs ≥ 10	3.61 (1.72‐7.57)	.001	4.77 (1.92‐11.87)	.001
<50 vs ≥ 50	9.48 (2.15‐41.83)	.003		
D‐dimer (<0.5 μg/mL vs ≥ 0.5 μg/mL)	2.84 (1.23‐6.56)	.015		
ALT (<50U/L vs ≥50 U/L)	3.68 (1.03‐13.11)	.045	6.87 (1.56‐30.21)	.011
NLR (<3.73 vs ≥3.73)	4.62 (1.91‐9.31)	<0.001		

Abbreviations: ALT, alanine transaminase; COPD, chronic obstructive lung disease; hs‐CRP, hypersensitive C‐reactive protein; NLR, neutrophil‐to‐lymphocyte ratio.

Further multivariate logistic regression analysis demonstrated chronic comorbidities (OR = 11.48, 95%CI: 4.44‐29.66, *P* = .001), elevated hs‐CRP (OR = 4.77, 95%CI:1.92‐11.87, *P* = .001), and elevated ALT (OR = 6.87, 95%CI: 1.56‐30.21, *P* = .011) were key risk factors for the tendency of severe phase. Diabetes (OR = 3.36, 95%CI: 1.41‐9.52, *P* = .008), hypertension (OR = 16.53, 95%CI: 2.07‐132.3, *P* = .008), and COPD (OR = 4.53, 95%CI: 1.21‐16.93, *P* = .025) all showed high risk while multivariate analysis was performed for the single comorbidity.

## 4 DISCUSSION

Our study provides some important insight into the differentiation of severe cases among the early COVID‐19 patients. The presence of chronic comorbidity is a key risk factor of developing into severe state. Recent study demonstrated that presence of hypertension, diabetes, COPD, and coronary artery disease were risk factors of disease progression in mild or moderate COVID‐19 patients, which is in accordance with our result.^6^ Moreover, patients with two or more comorbidities are prone to poor prognosis compared with those with no or single.^7^ The risk of severe predisposition in patients with chronic comorbidities is higher since it may be related to subsequent multiple organ function damage caused by SARS‐CoV‐2. Principally, increased gene expression of ACE‐2 in the airways of COPD patients may explain the susceptibility of SARS‐CoV‐2 and exacerbation of disease.^8^ Also, SARS‐CoV‐2 facilitated impaired insulin secretion through ACE2 in pancreatic endocrine cells, meanwhile diabetic patients are susceptible to viral infections, causing cytokine storms, and worsening clinical presentation.^9^

Our research suggested that the risk of severe illness increased with an elevated level of hs‐CRP. It has been noticed that dysregulation of inflammatory cytokines and chemokines contributes to severe COVID‐19. And the cytokine storm can induce apoptosis of endothelial cells and epithelial cells, which may cause vascular leakage and alveolar edema and lead to respiratory failure.^10^ CRP, stimulated by the release of cytokines, may reflect the severity of lung damage. Previous researchers have found that elevated hs‐CRP is related to respiratory function and lung lesions.^11^, ^12^ And recent evidence suggests that CRP was independently associated with the critical event‐free survival in patients with COVID‐19.^13^ CRP could be a promising biomarker for assessing disease lethality. And many researches in other countries, such as Singapore and Norway, have gotten the same results with us.^14^ Extensive research has shown that CRP might be a potential biomarker of response to IL‐6‐modulatory therapies for COVID‐19.^15^

Multivariate logistic regression analysis demonstrated that patients with an elevated level of ALT had a higher risk of exacerbation. It should be noted that the laboratory data used in our study were from early COVID‐19 patients, generally before medication. And some studies have found that abnormal levels of ALT were not associated with the liver condition.^16^ Thus, the elevated ALT is more likely the result of virus‐related liver cell injury instead of drug‐related cause or the liver condition of patients. At the same time, the liver histological examination of patients with COVID‐19 showed hepatocyte degeneration and necrosis, but no observed infection of SARS‐CoV‐2 in liver tissue.^17^ SARS‐CoV‐2 utilizes the angiotensin‐converting enzyme 2 (ACE2) as docking and entry receptor on host cells,^18^ but single cell RNA‐seq approaches indicated that ACE2 mRNA is expressed in bile duct cells, not or minimally in hepatocytes, and not in any other liver cell.^19^ Some researchers have point out that direct SARS‐CoV‐2 infection in hepatocytes and/or bile duct cells seems unlikely.^20^ On the other hand, COVID‐19 can cause excessive release of early response inflammatory factors,^21^ which may reflect cytokine storm syndrome and may be associated with multi‐organ failure (MOF). Pourya et al found that the average neutrophil percentage of patients with elevated ALT was significantly increased compared to patients with normal ALT, while the average lymphocyte percentage was significantly decreased.^22^ These evidences suggest that liver abnormalities in COVID‐19 patients may be due to systemic inflammatory response‐induced liver injuries and patients with early elevation of ALT may suffer the damage of cytokine storm and excessive immune response. Similarly, Bao et al also concluded in a meta‐analysis on 35 studies including 5912 COVID‐19‐positive patient that ALT could predict the progress of the COVID‐19 changes^14^ and many studies have demonstrated that severe and critical patients had a significantly higher incidence of abnormal ALT at admission.^23^

Older age, lymphocytopenia, higher NLR, and elevated D‐dimer were found to be the risk factors of severe tendency. Elder patients have presumably a stronger innate host immune response to SARS‐CoV‐2, which may lead to more serious pathological changes. Data from animal experiments suggested that aged macaques could develop a stronger immune response after being infected with SARS, which in turn can lead to more serious lung pathology.^24^ SARS‐CoV‐2, a virus similar to SARS, may have a similar pathological mechanism.

Lymphocytopenia is one of the most prominent features of patients with COVID‐19. Extensive researches have indicated that lymphocytopenia existed in all clinical types of COVID‐19 patients,^3^, ^25^, ^26^ especially in dead cases.^27^ The prognostic role of the NLR has been documented in different studies and NLR is an risk factor of in‐hospital mortality in COVID‐19 patients.^28^, ^29^ Recent evidence suggested that both T cells and NK cells in patients with COVID‐19 were reduced, and memory helper T cells and regulatory T cells were significantly decreased in severe patients.^30^ Our study also showed that lymphopenia and higher NLR were associated with the severity of the disease, but there was no further analysis of lymphocyte subsets due to the lack of relevant data. The autopsy report demonstrated that lymphocytes were overactivated despite the decreased number of lymphocytes.^31^ This suggests that SARS‐CoV‐2 may overactivate the immune system, destroy lymphocytes, and lead to the abnormal release of various cytokines. Consequently, these pathological changes may result in systemic hyperinflammation and indicate a poor prognosis.

A recent pathological work has established that patients with COVID‐19 revealed transparent thrombosis of microvessels.^17^ Abnormal blood coagulation was also observed in the course of the disease along with some obvious ischemic changes in certain dead patients.^17^, ^32^ Cui et al founded that patients with severe COVID‐19 could have an incidence of venous thromboembolism (VTE) of 25% and the D‐dimer is a good mark for predicting VTE.^33^ The increase D‐dimer in the early stage may reflect the abnormal blood coagulation function, the early appearance of pulmonary microthrombus, the rapid progression of the disease, and the transformation to severe cases.

Note that, such as other retrospective studies in commonplace, the clinical data of some patients may not be all inclusive; thus, the effect of other factors may be overlooked, such as coronary heart disease. Regarding the correlation between AST, ESR, LDH, and PCT and the prognosis of patients, the discrepancy from the earlier studies may be related to the different time of data collection.^3^, ^14^, ^25^ Our data were collected from the early stage of COVID‐19 patients due to the nature of our design inherent to this study. In addition, Ferritin is a key mediator of immune dysregulation contributing to the cytokine storm and recent researches demonstrated that elevated ferritin levels were related to disease severity and development of acute respiratory distress syndrome (ARDS),^34^, ^35^ which suggests that Ferritin might be a predictive factor for deterioration in COVID‐19 cases. Unfortunately, Ferritin was not measured in our patients and we could not analyze the influence of Ferritin on the early stage of COVID‐19. It is a limitation of our study. Finally, if this study were to include data from dead patients, a wider spectrum of potentially underestimated factors may be examined.

## 5 CONCLUSION

Early COVID‐19 patients with chronic comorbidities, elevated hs‐CRP or increased ALT are significantly more likely to develop severe pneumonia as disease progresses. If a patient's profile fits one of these three criteria, it will become highly important to raise priority for the critical care and surveillance of disease progress of the patient. Our findings may facilitate the early recognition of critical patients in clinical practice in dealing with this unprecedent COVID‐10 outbreak.

## CONFLICT OF INTEREST

There were no conflicts of interest to this work.

## AUTHOR CONTRIBUTIONS

Zhihao Xu conceived and designed the study. Jinrui Gao designed the study, analyzed the data, and wrote the first draft of the manuscript. Xiu Huang analyzed the data and wrote the first draft of the manuscript. Haibo Gu collected the clinical and CT data. Lingyun Lou did the analysis. All authors have read and approved the final manuscript and, therefore, have full access to all the data in the study and take responsibility for the integrity and security of the data.

## ETHICAL APPROVAL

This study was approved by the ethics committee of the Fourth Affiliated Hospital of Zhejiang University School of Medicine [K20200024]. All procedures followed were in accordance with the ethical standards of the responsible committee on human experimentation (institutional and national) and with the Helsinki Declaration of 1975, as revised in 2008(5). Informed consent was obtained from all patients for being included in the study.

## References

[1] Wilasang C , Sararat C , Jitsuk NC , et al. Reduction in effective reproduction number of COVID‐19 is higher in countries employing active case detection with prompt isolation. J Travel Med. 2020 27(5). 10.1093/jtm/taaa095 PMC7313773 32519743

[2] Pormohammad A , Ghorbani S , Khatami A , et al. Comparison of confirmed COVID‐19 with SARS and MERS cases ‐ Clinical characteristics, laboratory findings, radiographic signs and outcomes: A systematic review and meta‐analysis. Rev Med Virol. 2020 30(4):e2112. 32502331 10.1002/rmv.2112 PMC7300470

[3] Huang C , Wang Y , Li X , et al. Clinical features of patients infected with 2019 novel coronavirus in Wuhan, China. Lancet. 2020;395(10223):497‐506. 31986264 10.1016/S0140-6736(20)30183-5 PMC7159299

[4] Wu Z , McGoogan JM . Characteristics of and Important Lessons From the Coronavirus Disease 2019 (COVID‐19) Outbreak in China: Summary of a Report of 72 314 Cases From the Chinese Center for Disease Control and Prevention. JAMA. 2020;323(13):1239. 32091533 10.1001/jama.2020.2648

[5] Liu Y , Yan LM , Wan L , et al. Viral dynamics in mild and severe cases of COVID‐19. Lancet Infect Dis. 2020;6:656‐657. 10.1016/S1473-3099(20)30232-2 PMC7158902 32199493

[6] Cen Y , Chen X , Shen Y , et al. Risk factors for disease progression in mild to moderate COVID‐19 patients‐ a multi‐center observational study. Clin Microbiol Infect. 2020;26(9):1242–1247. 10.1016/j.cmi.2020.05.041 PMC7280135 32526275

[7] Guan WJ , Liang WH , Zhao Y , et al. Comorbidity and its impact on 1590 patients with COVID‐19 in China: a nationwide analysis. Eur Respir J. 2020;55(5). 10.1183/13993003.0054-2020 PMC7098485 32217650

[8] Leung JM , Yang CX , Tam A , et al. ACE‐2 expression in the small airway epithelia of smokers and COPD patients: implications for COVID‐19. Eur Respir J. 2020;55(5):2000688. 32269089 10.1183/13993003.00688-2020 PMC7144263

[9] Maddaloni E , Buzzetti R . Covid‐19 and diabetes mellitus: unveiling the interaction of two pandemics. Diabetes Metab Res Rev. 2020;e33213321 10.1002/dmrr.3321 32233018 PMC7228318

[10] Ye Q , Wang B , Mao J . The pathogenesis and treatment of the `Cytokine Storm' in COVID‐19. J Infect. 2020;80(6):607‐613. 32283152 10.1016/j.jinf.2020.03.037 PMC7194613

[11] Wang L . C‐reactive protein levels in the early stage of COVID‐19. Med Mal Infect. 2020;50(4):332‐334. 32243911 10.1016/j.medmal.2020.03.007 PMC7146693

[12] Erika P , Domenica Z , Paolo I , et al. Lactate dehydrogenase and C‐reactive protein as predictors of respiratory failure in CoVID‐19 patients. Clin Chim Acta. 2020;509:135‐138. 32531257 10.1016/j.cca.2020.06.012 PMC7282743

[13] Park B , Park J , Lim JK , et al. Prognostic Implication of Volumetric Quantitative CT Analysis in Patients with COVID‐19: A Multicenter Study in Daegu, Korea. Korean Journal of Radiology. 2020;21:e130. 10.3348/kjr.2020.0567 PMC7462758 32767868

[14] Bao J , Li C , Zhang K , Kang H , Chen W , Gu B . Comparative analysis of laboratory indexes of severe and non‐severe patients infected with COVID‐19. Clin Chim Acta. 2020;509:180‐194. 32511971 10.1016/j.cca.2020.06.009 PMC7274996

[15] Montesarchio V , Parella R , Iommelli C , et al. Outcomes and biomarker analyses among patients with COVID‐19 treated with interleukin 6 (IL‐6) receptor antagonist sarilumab at a single institution in Italy. J Immunother Cancer. 2020;8(2):e001089. 32784217 10.1136/jitc-2020-001089 PMC7418768

[16] Gu X , Li X , An X , et al. Elevated serum aspartate aminotransferase level identifies patients with coronavirus disease 2019 and predicts the length of hospital stay. J Clin Lab Anal. 2020;34(7):e23391. 32488888 10.1002/jcla.23391 PMC7300531

[17] Yao XH , Li TY , He ZC , et al. A pathological report of three COVID‐19 cases by minimal invasive autopsies. Zhonghua Bing Li Xue Za Zhi. 2020;49(5):411‐417. 32172546 10.3760/cma.j.cn112151-20200312-00193

[18] Zhou P , Yang XL , Wang XG , et al. A pneumonia outbreak associated with a new coronavirus of probable bat origin. Nature. 2020;579(7798):270‐273. 32015507 10.1038/s41586-020-2012-7 PMC7095418

[19] Qi F , Qian S , Zhang S , Zhang Z . Single cell RNA sequencing of 13 human tissues identify cell types and receptors of human coronaviruses. Biochem Biophys Res Commun. 2020;526(1):135‐140. 32199615 10.1016/j.bbrc.2020.03.044 PMC7156119

[20] Bertolini A , van de Peppel IP , Bodewes F , et al. Abnormal liver function tests in COVID‐19 patients: relevance and potential pathogenesis. Hepatology (Baltimore, MD). 2020 10.1002/hep.31480 PMC7404414 32702162

[21] Liu J , Li S , Liu J , et al. Longitudinal characteristics of lymphocyte responses and cytokine profiles in the peripheral blood of SARS‐CoV‐2 infected patients. EBioMedicine. 2020;55:102763. 32361250 10.1016/j.ebiom.2020.102763 PMC7165294

[22] Gholizadeh P , Safari R , Marofi P , et al. Alteration of Liver Biomarkers in Patients with SARS‐CoV‐2 (COVID‐19). J Inflamm Res. 2020;13:285‐292. 32669866 10.2147/JIR.S257078 PMC7335895

[23] Wu Y , Li H , Guo X , et al. Incidence, risk factors, and prognosis of abnormal liver biochemical tests in COVID‐19 patients: a systematic review and meta‐analysis. Hepatol Int. 2020;1‐17. 32710250 10.1007/s12072-020-10074-6 PMC7380163

[24] Smits SL , de Lang A , van den Brand JM , et al. Exacerbated innate host response to SARS‐CoV in aged non‐human primates. PLoS Pathog. 2010;6(2):e1000756. 20140198 10.1371/journal.ppat.1000756 PMC2816697

[25] Zhang JJ , Dong X , Cao YY , et al. Clinical characteristics of 140 patients infected with SARS‐CoV‐2 in Wuhan, China. Allergy. 2020;75(7):1730‐1741. 32077115 10.1111/all.14238

[26] Xu T , Chen C , Zhu Z , et al. Clinical features and dynamics of viral load in imported and non‐imported patients with COVID‐19. Int J Infect Dis. 2020;94:68‐71. 32179140 10.1016/j.ijid.2020.03.022 PMC7270709

[27] Zhou F , Yu T , Du R , et al. Clinical course and risk factors for mortality of adult inpatients with COVID‐19 in Wuhan, China: a retrospective cohort study. Lancet. 2020;395(10229):1054‐1062. 32171076 10.1016/S0140-6736(20)30566-3 PMC7270627

[28] Liu Y , Du X , Chen J , et al. Neutrophil‐to‐lymphocyte ratio as an independent risk factor for mortality in hospitalized patients with COVID‐19. J Infect. 2020;81(1):e6‐e12. 10.1016/j.jinf.2020.04.002 PMC7195072 32283162

[29] Lian J , Jin C , Hao S , et al. High neutrophil‐to‐lymphocyte ratio associated with progression to critical illness in older patients with COVID‐19: a multicenter retrospective study. Aging. 2020;12(14):13849‐13859. 32730223 10.18632/aging.103582 PMC7425510

[30] Qin C , Zhou L , Hu Z , et al. Dysregulation of immune response in patients with COVID‐19 in Wuhan, China. Clin Infect Dis. 2020;71(15):762–768. 32161940 10.1093/cid/ciaa248 PMC7108125

[31] Xu Z , Shi L , Wang Y , et al. Pathological findings of COVID‐19 associated with acute respiratory distress syndrome. Lancet Respir Med. 2020;8(4):420‐422. 32085846 10.1016/S2213-2600(20)30076-X PMC7164771

[32] Li T , Lu H , Zhang W . Clinical observation and management of COVID‐19 patients. Emerg Microbes Infect. 2020;9(1):687‐690. 32208840 10.1080/22221751.2020.1741327 PMC7103696

[33] Cui S , Chen S , Li X , Liu S , Wang F . Prevalence of venous thromboembolism in patients with severe novel coronavirus pneumonia. J Thromb Haemost. 2020;18(6):1421‐1424. 32271988 10.1111/jth.14830 PMC7262324

[34] Bellmann‐Weiler R , Lanser L , Barket R , et al. Prevalence and Predictive Value of Anemia and Dysregulated Iron Homeostasis in Patients with COVID‐19 Infection. J Clin Med. 2020;9(8):2429. 10.3390/jcm9082429 PMC7464087 32751400

[35] Wu C , Chen X , Cai Y , et al. Risk factors associated with acute respiratory distress syndrome and death in patients with coronavirus disease 2019 pneumonia in Wuhan, China. JAMA Intern Med. 2020;180(7):934‐943. 32167524 10.1001/jamainternmed.2020.0994 PMC7070509

